# Increased Frequency of Diabetic Ketoacidosis: The Link With COVID-19 Pandemic

**DOI:** 10.3389/fcdhc.2022.846827

**Published:** 2022-02-24

**Authors:** Giuseppe d’Annunzio, Marta Bassi, Elena Lucia De Rose, Marilea Lezzi, Nicola Minuto, Maria Grazia Calevo, Alberto Gaiero, Graziella Fichera, Riccardo Borea, Mohamad Maghnie

**Affiliations:** ^1^ Pediatric Clinic and Endocrinology Unit, Regional Center for Pediatric Diabetes, IRCCS Istituto Giannina Gaslini, Genoa, Italy; ^2^ Department of Pediatrics, Istituto di Ricovero e Cura a Carattere Scientifico (IRCCS) Istituto Giannina Gaslini, Genoa, Italy; ^3^ Department of Neuroscience, Rehabilitation, Ophthalmology, Genetics and Maternal Child Health, University of Genoa, Genoa, Italy; ^4^ Epidemiology and Biostatistics Unit, Istituto di Ricovero e Cura a Carattere Scientifico (IRCCS) Istituto Giannina Gaslini, Genoa, Italy; ^5^ Pediatric and Neonatology Unit, San Paolo Hospital, Savona, Italy; ^6^ Pediatric Unit, Sanremo Hospital, Sanremo, Italy

**Keywords:** type 1 diabetes mellitus, diabetic ketoacidosis, COVID-19, pandemic, health care services, children

## Abstract

**Aims:**

Diabetic ketoacidosis is the most severe metabolic derangement due to prolonged insulin deficiency as in type 1 diabetes. Diabetic ketoacidosis, a life-threatening condition, is often diagnosed late. A timely diagnosis is mandatory to prevent its consequences, mainly neurological. The COVID-19 pandemic and lockdown have reduced the availability of medical care and access to hospitals. The aim of our retrospective study was to compare the frequency of ketoacidosis at the diagnosis of type 1 diabetes between the lockdown-post lockdown period and the previous two calendar years, in order to evaluate the impact of the COVID-19 pandemic.

**Patients and Methods:**

We retrospectively assessed the clinical and metabolic data at the diagnosis of type 1 diabetes in children in the Liguria Region during 3 different time periods: calendar year 2018 (Period A), calendar year 2019 until February 23,2020 (Period B) and from February 24, 2020 onwards to March 31, 2021 (Period C).

**Results:**

We analyzed 99 patients with newly-diagnosed T1DM from 01/01/2018 to 31/03/2021. Briefly, a younger age at diagnosis of T1DM was observed in Period 2 compared to Period 1 (p = 0.03). The frequency of DKA at clinical onset of T1DM was similar in Period A (32.3%) and Period B (37.5%), while it significantly increased in Period C (61.1%) compared to Period B (37.5%) (p = 0.03). PH values were similar in Period A (7.29 ± 0.14) and Period B (7.27 ± 0.17), while they were significantly lower in Period C (7.21 ± 0.17) compared to Period B (p = 0.04).

**Conclusions:**

An increase in the frequency of diabetic ketoacidosis has been documented in newly diagnosed pediatric patients in the Liguria Region during and after the lockdown period compared to previous calendar years. This increase could have been caused by the delay in diagnosis following the restrictions imposed by the lockdown with consequently reduced access to health care facilities. More information on the risks of ketoacidosis is desirable by means of social and medical awareness campaigns.

## Introduction

In early December 2019, the first patient suffering from a strange form of pneumonia due to a new infectious agent was reported in Hubei Province, China (1). The disease caused by the Severe Respiratory Syndrome Corona Virus 2 (SARS-CoV-2), recognized as Coronavirus Disease 19 (COVID-19) has spread rapidly across China and worldwide. The World Health Organization defined the COVID-19 outbreak a Public Health Emergency of International Concern on January 30, 2020, and a pandemic was declared on March 11, 2020. On March 9 an epidemic state was declared and a subsequent lockdown restriction was imposed by the government from March 9 to May 3, 2020 (2). These measures were followed by a reduction in new cases of COVID-19 infection, which intensely modified the lifestyle of healthy and sick people.

In addition to different and conflicting data reported on its clinical severity, the COVID-19 pandemic has represented a serious global concern for patients with chronic conditions, as outpatient activities have been suspended to address emergency situations, with consequent impairment of follow-up programs. Moreover, the restrictions imposed by the lockdown together with the fear of being infected have reduced the number of accesses and the availability of health care services. In addition, the resources and workforce were inevitably concentrated on the COVID-19 pandemic. As a result, attention to diseases other than COVID-19 has decreased, with subsequent delay in the recognition and diagnosis of several serious pediatric diseases, such as type 1 diabetes mellitus (3).

Type 1 diabetes mellitus (T1DM) is an autoimmune disease that occurs in genetically susceptible subjects. Genetic susceptibility alone does not explain the development of the disease, therefore environmental factors play a role as a trigger for the autoimmune response (4, 5). Although several factors have been studied, a specific one is not yet known (5, 6).

The clinical onset of T1DM is preceded by an asymptomatic period characterized by insulitis responsible for progressive insulin deficiency, with loss of glucose availability (7). The consequences of insulin deficiency are the rise of counter-regulatory hormones, blood glucose levels and osmolality, resulting in polyuria and polydipsia up to a metabolic decompensation called Diabetic Ketoacidosis (DKA) (8).

DKA is a severe complication of T1DM caused by an insulin deficiency. It includes hyperglycemia affecting osmotic diuresis and volume depletion, electrolytes imbalance, production of ketoacids, and metabolic acidosis. Weight loss, polyuria, and polydipsia are reported. DKA, whose severity depends on delayed recognition, is observed not only at clinical diagnosis of T1DM but also during follow-up, and represents a seriously negative prognostic factor (9, 10).

According to the ISPAD Guidelines, DKA is defined as random plasma glucose >250 mg/dl, pH <7.3, and serum bicarbonate <15 mEq/l (11).

The worldwide incidence of T1DM is variable but has grown more than two to three times in recent decades, particularly in Finland and in the Sardinia region, Italy (12, 13). Similarly, DKA shows an even higher prevalence than expected, despite the periodic implementation of awareness campaigns (14). In Italy, DKA accounts for up to 41.2% of new cases of T1DM (12). DKA can also develop in patients with well-known T1DM; it is referred to as recurrent DKA, and its frequency varies among different countries, reaching up to 8 per 100 person-years (15). Correct and timely diagnosis of T1DM and also careful follow-up of patients are the cornerstones for DKA prevention, which should be a goal for pediatricians (16, 17). Several data records report an increased frequency of DKA during the COVID-19 pandemic (18–22). Reduced hospital admissions and increased fear of COVD-19 have been considered the most important factors.

The aim of our cross-sectional study was to compare the frequency of DKA between different time periods, i.e., before and during the lockdown period due to the COVID-19 pandemic.

## Materials and Methods

We retrospectively analyzed clinical and metabolic data of all newly-diagnosed T1DM patients, aged 0.18 years, in the calendar years of 2018 to 2021. Data were collected from the medical records of 3 different pediatric hospitals active in the Liguria Region: the Giannina Gaslini Pediatric Clinic, the hub hosting the Regional Reference Center for Pediatric Diabetes established according to the Regional Law n. 27, 9 August 2013 (23), and 2 spokes: the Pediatric and Neonatology Unit, San Paolo Hospital, Savona and the Maternal Childhood Department, Imperia Hospital.

We enrolled 99 children and adolescents living in the Liguria Region, diagnosed during 3 different time periods: calendar year 2018 (Period A) and calendar year 2019 until February 23,2020 (Period B), i.e., before the spread of the COVID-19 pandemic, and from February 24, 2020 to March 31, 2021 (Period C), i.e., during the pandemic period.

Period A included 31 patients, Period B included 32 patients and Period C included 36 patients. Mean age was 10.3 ± 4.4 years in Period A, 8.2 ± 3.7 years in Period B and 8.8 ± 4.7 years in Period C. Clinical characteristics are reported in **Table 1**. T1DM was diagnosed according to the ISPAD Guidelines, i.e., random glucose levels >250 mg/dl, in the presence of symptoms such as polyuria, polydipsia, and weight loss up to DKA. Severity of DKA was classified according to the ISPAD Guidelines: random plasma glucose >250 mg/dl, pH <7.3, and serum bicarbonate <15 mEq/l. In particular, 7.1 ≤pH <7.3 defines mild/moderate DKA and pH <7.1 defines severe DKA (11).

**Table 1:** Clinical characteristics of the enrolled patients.

Time period	2018	January 1, 2019 to February 23, 2020	February 24, 2020 to March 31, 2021	p-value 2018 vs 2019	p-value2019 vs 2020	p-value2018 vs 2020
	PERIOD A	PERIOD B	PERIOD C			
	N = 31	N = 32	N = 36			
Age at T1DM diagnosis (years)	10.30 ± 4.44	8.24 ± 3.66	8.77 ± 4.70	**0.03**	0.78	0.11
HbA1c (%)	11.07 ± 2.9	11.04 ± 1.9	10.8 ± 2	0.81	0.61	0.90
BMI-SDS	−0.20 ± 1.07	0.11 ± 1.66	0.21 ± 1.17	0.69	0.57	0.25
	**N (%)**	**N (%)**	**N (%)**			
Celiac disease (tTGAAb)	2 (9.5)	5 (20)	7 (24.1)	0.43	0.75	0.27
TgAb	1 (5.6)	1 (4.5)	3 (10.7)	1	0.62	1
TPOAb	2 (11.1)	1 (4.5)	1 (3.6)	0.58	1	0.55
DKA	10 (32.3)	12 (37.5)	22 (61.1)	0.79	0.09	**0.03**
pH	7.29 ± 0.14	7.27 ± 0.17	7.21 ± 0.17	0.80	0.06	**0.04**
pH*						
<7	2 (6.9)	3 (9.4)	7 (20.6)			
≥7 e <7.3	8 (27.6)	9 (28.1)	15 (44.1)			
≥7.3	19 (65.5)	20 (62.5)	12 (35.3)			

*p-value = 0.09. Bold means statistical significant.

The positivity of one or more markers of β-cell autoimmunity, i.e., anti-Glutamic Acid Decarboxylase (GAD), anti insulin (IA), anti Zinc Transporter 8 (ZnT8), anti-Tyrosine Phosphatase 2 (IA2) autoantibodies confirmed the autoimmune etiology of diabetes. Patients with type 2 diabetes, monogenic or other types of diabetes, and other forms of secondary dysglycemia were excluded from the study.

For each T1DM patient we evaluated height with Harpender stadiometer to the nearest 0.1 cm, weight to the nearest 0.1 kg with a calibrated scale, BMI calculated as weight in kilograms divided by the square of height in meters. BMI-SDS was calculated on the reference values of age and gender. We also measured random plasma glucose, HbA1c, fasting C-peptide, total serum IgA levels, liver and kidney function, and serum markers of autoimmune diseases frequently associated to T1DM, i.e., celiac disease by means of anti-transglutaminase IgA (tTGAAb), and thyroid autoimmune disease by means of anti-thyroglobulin (TgAb) and thyroperoxidase (TPOAb) autoantibodies, and serum levels of FT4 and TSH.

Nasal swab for the diagnosis of COVID-19 infection was performed in all patients admitted during and after the lockdown period.

Our study analyzed anonymized and unidentifiable clinical data collected routinely at diabetes clinical onset in the medical records from three hospitals involved, and would not affect care of patients, therefore we consider ethical committee approval to be not mandatory.

### Statistical Analysis

Descriptive statistics were generated for the entire cohort and data were expressed as mean and standard deviation (SD), for continuous variables, and as absolute or relative frequencies for categorical variables. Non-parametric analysis (Mann–Whitney U-test) for continuous variables and the Chi square or Fisher’s exact test for categorical variables were used to measure differences between groups. P-values ≤0.05 were considered statistically significant, and all P-values were based on two tailed tests. Statistical analysis was performed using SPSS for Windows (SPSS Inc., Chicago, IL, USA).

## Results

We analyzed 99 patients with newly-diagnosed T1DM from January 1, 2018 to March 31, 2021. Clinical and metabolic data are reported in **Table 1**.

DKA was present in 44% of newly-diagnosed patients. In particular we observed 10 cases (32.3%) of DKA in Period A, 12 cases (37.5%) in Period B and 22 cases (61.1%) in Period C. The frequency of DKA at T1DM clinical onset was similar in Period A patients (32.3%) and Period B patients (37.5%), while it significantly increased in Period C patients (61.1%) compared to Period B patients (37.5%) (p = 0.03) (**Table 1**).

Briefly, a younger age at disease diagnosis was observed in Period B patients compared to Period A patients (p = 0.03) (**Table 1**). No significant differences were found in age at diagnosis between Period B patients and Period C patients.

Moreover, pH values were similar in Period A patients (7.29 ± 0.14) and in Period B patients (7.27 ± 0.17), while they were significantly lower in Period C patients (7.21 ± 0.17) compared to Period B patients (p = 0.04) (**Table 1**).

HbA1c levels and BMI-SDS were not different between the 3 calendar periods patients. Similarly, we did not found any difference in the frequency of celiac disease and/or thyroid autoimmune diseases in our patients. Nasal swab for COVID-19 was negative in all tested patients and their caregivers.

During this time period only a girl admitted with mild metabolic decompensation, obesity, and family history for type 2 diabetes mellitus and absence of β-cell autoimmunity markers was diagnosed as type 2 diabetes and was not included in this study.

## Discussion

In our retrospective study we evaluated the different frequencies of DKA at T1DM clinical diagnosis in a population of children and adolescents referred: 1 our Regional Reference Center for Pediatric Diabetes and 2 pediatric hospital in-ward in the Liguria Region. Our findings provide an additional picture of the clinical changes that occurred during the COVID-19 pandemic in Italy regarding T1DM, and confirm the increased frequency DKA in newly-diagnosed pediatric T1DM, as reported by others (18).

DKA still represents the worst clinical onset of T1DM and is an acute and life-threatening complication of the disease. DKA negatively affects the entire course of the disease, being associated with longer hospitalization, more difficult achievement of good metabolic control, reduced frequency and duration of the remission phase, even higher morbidity and mortality rate (9).

Several conditions have been evaluated as predisposing factors for DKA, namely, younger childhood, absence of first-degree relatives with T1DM, low socioeconomic status (meaning less availability of medical care), and poor awareness of diabetes symptoms (17). The increased severity of and frequency of DKA at T1DM clinical onset reported during and after lockdown period could be the consequence of a substantial decrease in attendance at the Pediatric Emergency Units and general practitioners. The risk of delayed diagnosis of several potentially serious conditions, such as T1DM, has been reported in both the adult and pediatric age groups (24, 25). A possible explanation for this delay in diagnosis could be the increased attention of healthcare system to the COVD-19 pandemic and related problems, together with the difficulties of travel and reduced access to medical care for children and their families linked to the lockdown itself. It is noteworthy that delayed diagnosis and scarce awareness are the most important factors for DKA severity.

An Italian pediatric multicenter cross-sectional study aimed at evaluating whether the initial phase of COVID-19 pandemic influenced the frequency and severity of DKA recorded data from 68 pediatric diabetes Centers belonging to the Italian Society for Pediatric Endocrinology and Diabetes (18). The authors reported a 23% reduction in T1DM cases in 2020 compared to 2019. However, among patients diagnosed with DKA, the frequency of severe DKA was higher in 2020 than in 2019 (18). The authors concluded that the early clinical diagnosis of T1DM was severely affected by the pandemic, and that greater awareness is warranted in case of a “second wave”. It is worth noting that we did not observe a reduction in the number of new T1DM cases in our case series. Another study evaluated the frequency of DKA in 532 pediatric patients with newly-diagnosed T1DM using data from the German Diabetes Prospective Follow-up Registry, which covers more than 90% of pediatric T1DM. DKA was observed in 44.7% of cases and severe DKA in 19.4% of cases, with a significantly higher frequency than in the 2 previous years (19). The authors hypothesized multifactorial underlying causes, notably the reduction of medical services and the fear of approaching the health care systems, and socioeconomic factors (24, 25).

The incidence of T1DM in 2020 has been reported to follow the upward trend reported between 2011 and 2019, indicating the lack of short-term influence of the COVID-19 pandemic. Despite the stressful situation, social distancing during the lockdown reduced the exposure to common infections, which are a trigger for the development of DKA (20).

The UK Association of Children’s Diabetes Clinicians found a 51% DKA rate at T1DM clinical diagnosis, higher than previously observed, and ascribed to fear of COVID-19, inability to access medical services, and misdiagnosed symptoms as the most important causative factors (21).

A Polish study reported a 12% higher incidence of DKA in pediatric patients diagnosed withT1DM in 2020 compared to 2019, with a significant increase in DKA severity (22).

Type 1 and type 2 diabetes increase the negative impact of COVID-19 infection (26), therefore patients with T1DM deserve attention (27). To this purpose, recommendations for COVID-19 in children and adolescents with T1DM have been published (28). Despite the increased frequency of DKA at T1DM clinical onset, no association with other autoimmune diseases has been found (29, 30).

We are aware that our study confirms previous findings already reported, otherwise it strengthens the importance of a global awareness campaign aimed to avoid delayed diagnosis and the subsequent risk of DKA.

Our study has several limitations. First of all, while dealing with a very serious typical issue for pediatric diabetes, it confirms the results already reported. Moreover, the sample size is not so relevant to be considered representative of a population-based study. Further epidemiological studies that also take into account the impact of socioeconomic status on the severity of T1DM are mandatory.

## Data Availability Statement

The original contributions presented in the study are included in the article/supplementary material. Further inquiries can be directed to the corresponding author.

## Ethics Statement

Ethical review and approval was not required for the study on human participants in accordance with the local legislation and institutional requirements. Written informed consent to participate in this study was provided by the participants’ legal guardian/next of kin. Written informed consent was not obtained from the individual(s), or the minor(s)’ legal guardian/next of kin, for the publication of any potentially identifiable images or data included in this article.

## Author Contributions

Gd’A conceptualized and wrote the paper. MB, ELDR, and ML collected data. NM, AG, GF, and RB followed the patients and collected data. MGC performed statistical analysis. MM revised and approved the manuscript. All authors listed have made a substantial, direct, and intellectual contribution to the work and approved it for publication.

## Conflict of Interest

The authors declare that the research was conducted in the absence of any commercial or financial relationships that could be construed as a potential conflict of interest.

## Publisher’s Note

All claims expressed in this article are solely those of the authors and do not necessarily represent those of their affiliated organizations, or those of the publisher, the editors and the reviewers. Any product that may be evaluated in this article, or claim that may be made by its manufacturer, is not guaranteed or endorsed by the publisher.

## References

[1] Statement on the Second Meeting of the International Health Regulations. Emergency Committee Regarding the Outbreak of Novel Coronavirus (2019-Ncov). World Health Organization (WHO) (Press Release). 30th January 2020. Archived From the Original on 31st January 2020. Retrieved 30th January 2020 (2005). Available at: https://www.who.int/news-room/detail/30-01-2020-statement-on-the-secondmeeting-of-the-international-health-regulations-(2005)-emergency-committee-regarding-the-outbreak-of-novelcoronavirus-(2019-ncov) (Accessed July 30, 2020).

[2] WHO Director-General’s Opening Remarks at the Briefingon COVID-19 – 11th March 2020. World Health Organization (WHO) (Press Release). 11th March 2020. Achieved From the Original on 11th March 2020. Retrieved12th March 2020. Available at: https://www.who.int/dg/speeches/detail/who-director-general-s-opening-remarks-at-the-mediabriefing-on-covid-19—11-march-2020 (Accessed July 30,2020).

[3] DiMeglio LA Evans-Molina C Oram RA. Type 1 Diabetes. Lancet (2018) 16;391(1.0138):2449–62. doi: 10.1016/S0140-6736(18)31320-5 PMC6661119 29916386

[4] Craig ME Kim KW Isaacs SR Penno MA Hamilton-Williams EE Couper JJ. Early-Life Factors Contributing to Type 1 Diabetes. Diabetologia (2019) 62:1823–34. doi: 10.1007/s00125-019-4942-x 31451871

[5] Ying X Xie Z Huang G Zhou Z. Incidence and Trend of Type 1 Diabetes and the Underlying Environmental Determinants. Diabetes Metab Res Rev (2019) 35:e3075. doi: 10.1002/dmrr.3075 30207035

[6] Dedrick S Sundaresh B Huang Q Brady C Yoo T Cronin C. The Role of Gutmicrobiotaand Environmental Factors in Type 1 Diabetespathogenesis. Front Endocrinol (Lausanne) (2020) 26,11:78. doi: 10.3389/fendo.2020.00078 PMC7057241 32174888

[7] Atkinson MA Eisenbarth GS Michels AW. Type 1 Diabetes. Lancet (2014) 4;383(9911):69–82. doi: 10.1016/S0140-6736(13)60591-7 PMC4380133 23890997

[8] Cashen K Petersen T. Diabetic Ketoacidosis. Pediatr Rev (2019) 40(8):412–20. doi: 10.1542/pir.2018-0231.PMID:31371634 31371634

[9] Realsen J Goettle H Chase HP. Morbidity and Mortality of Diabeticketoacidosis With and Without Insulin Pump Care. Diabetes Technol Ther (2012) 14(12):1149–54. doi: 10.1089/dia.2012.0161 23009106

[10] Malik FS Hall M Mangione-Smith R Keren R Mahant S Shah SS. Patient Characteristics Associated With Differences in Admission Frequency for Diabetic Ketoacidosis in United States Children’s Hospitals. J Pediatr (2016) 171:104–10. doi: 10.1016/j.jpeds.2015.12.015 26787380

[11] Wolfsdorf JI Glaser N Agus M Fritsch M Hanas R Rewers A. ISPAD Clinical Practice Consensus Guidelines 2018: Diabetic Ketoacidosis and the Hyperglycemic Hyperosmolar State. Pediatr Diabetes (2018) 19(Suppl 27):155–77. doi: 10.1111/pedi.12701 29900641

[12] Cherubini V Grimsmann JM Åkesson K Birkebæk NH Cinek O Dovč K. Temporal Trends in Diabetic Ketoacidosis at Diagnosis of Paediatric Type 1 Diabetes Between 2006 and 2016: Results From 13 Countries in Three Continents. Diabetologia (2020) 63(8):1530–41. doi: 10.1007/s00125-020-05152-1 PMC7351855 32382815

[13] Songini M Mannu C Targhetta C Bruno G. Type 1 Diabetes in Sardinia: Facts and Hypotheses in the Context of Worldwide Epidemiological Data. Acta Diabetol (2017) 54(1):9–17. doi: 10.1007/s00592-016-0909-2 27639869

[14] Rabbone I Maltoni G Tinti D Zucchini S Cherubini V Bonfanti R. Diabetic Ketoacidosis at the Onset of Disease During a National Awareness Campaign: A 2-Year Observational Study in Children Aged 0-18 Years. Arch Dis Child (2020) 105(4):363–6. doi: 10.1136/archdischild-2019-316903 31597646

[15] Garibaldi L Becker D. Is the Risk of Diabetic Ketoacidosis Modifiable? J Pediatr (2016) 171:10–2. doi: 10.1016/j.jpeds.2016.01.057 26879811

[16] Iovane B Cangelosi AM Bonaccini I Di Mauro D Scarabello C Panigari A. Diabetic Ketoacidosis at the Onset of Type 1 Diabetes in Young Children: Is It Time to Launch a Tailored Campaign for DKA Prevention in Children <5 Years? Acta BioMed (2018) 8;89:67–71. doi: 10.23750/abm.v89i1.6936 29633745 PMC6357617

[17] Gesuita R Maffeis C Bonfanti R Cardella F Citriniti F d’Annunzio, G. et al. Network of the Italian Society of Pediatric Endocrinology and Diabetes (ISPED) for DKA Study and Prevention. Socioeconomic Inequalities Increase the Probability of Ketoacidosis at Diagnosis of Type 1 Diabetes: A 2014-2016 Nationwide Study of 2,679 Italian Children. Front Pediatr (2020) 22;8:575020:575020. doi: 10.3389/fped.2020.575020 PMC7642455 33194905

[18] Rabbone I Schiaffini R Cherubini V Maffeis C Scaramuzza A Diabetes Study Group of the Italian Society for Pediatric Endocrinology and Diabetes. Has COVID-19 Delayed the Diagnosis and Worsened the Presentation of Type 1 Diabetes in Children? Diabetes Care (2020) 43:2870–2. doi: 10.2337/dc20-1321 32778554

[19] Kamrath C Mönkemöller K Biester T Rohrer TR Warncke K Hammersen J. Ketoacidosis in Children and Adolescents With Newly Diagnosed Type 1 Diabetes During the COVID-19 Pandemic in Germany. JAMA (2020) 324(8):801–4. doi: 10.1001/jama.2020.13445 PMC7372511 32702751

[20] Tittel SR Rosenbauer J Kamrath C Ziegler J Reschke F Hammersen J. Did the COVID-19 Lockdown Affect the Incidence of Pediatric Type 1 Diabetes in Germany? Diabetes Care (2020) 43(11):e172–3. doi: 10.2337/dc20-1633 PMC7576433 32826282

[21] Ng SM Woodger K Regan F Soni A Wright N Agwu JC. Presentation of Newly Diagnosed Type 1 Diabetes in Children and Young People During COVID-19: A National UK Survey. BMJ Paediatr Open (2020) 2;4(1):e000884. doi: 10.1136/bmjpo-2020-000884 PMC7607512 34192183

[22] Dżygało K Nowaczyk J Szwilling A Kowalska A. Increased Frequency of Severe Diabetic Ketoacidosis at Type 1 Diabetes Onset Among Children During COVID-19 Pandemic Lockdown: An Observational Cohort Study. Pediatr. Endocrinol Diabetes Metab (2020) 26(4):167–75. doi: 10.5114/pedm.2020.101003 33554490

[23] Legge Regionale 27 Agosto 2013, N. 27. Norme Per La Prevenzione, La Diagnosi E La Cura Del Diabete Mellito (Bollettino Ufficiale N. 14, Del 14.08.2013) Genova (2013). Available at: https://www.siditalia.it/pdf/PIANO%20MALATTIA%20DIABETICA%20LIGURIA%20legge_2013_27_v1.pdf.

[24] Lazzerini M Barbi E Apicella A Marchetti F Cardinale F Trobia G. Delayed Access or Provision of Care in Italy Resulting From COVID-19. Lancet Child Adolesc Health (2020) 4(5):e10–1. doi: 10.1016/S2352-4642(20)30108-5 PMC7146704 32278365

[25] Cella A Marchetti F Iughetti L Di Biase AR Graziani G De Fanti A. Italian COVID-19 Epidemic: Effects on Paediatric Emergency Attendance-A Survey in the Emilia Romagna Region. BMJ Paediatr Open (2020) 20;4(1):e000742. doi: 10.1136/bmjpo-2020-000742 PMC10577789 34192169

[26] Gregory JM Slaughter JC Duffus SH Smith TJ LeStourgeon LM Jaser SS. COVID-19 Severity Is Tripled in the Diabetes Community: A Prospective Analysis of the Pandemic’s Impact in Type 1 and Type 2 Diabetes. Diabetes Care (2021) 44(2):526–32. doi: 10.2337/dc20-2260 PMC7818316 33268335

[27] Iughetti L Trevisani V Cattini U Bruzzi P Lucaccioni L Madeo S. COVID-19 and Type 1 Diabetes: Concerns and Challenges. Acta BioMed (2020) 7;91(3):e2020033. doi: 10.23750/abm.v91i3.10366 PMC7716973 32921727

[28] d’Annunzio G Maffeis C Cherubini V Rabbone I Scaramuzza A Schiaffini R. Caring for Children and Adolescents With Type 1 Diabetes Mellitus: Italian Society for Pediatric Endocrinology and Diabetology (ISPED) Statements During COVID-19 Pandemia. Diabetes Res Clin Pract (2020) 168:108372. doi: 10.1016/j.diabres.2020.108372 32827594 PMC7438223

[29] Parkkola A Härkönen T Ryhänen SJ Uibo R Ilonen J Knip M. Transglutaminase Antibodies and Celiacdisease in Children With Type 1 Diabetes and in Their Family Members. Pediatr Diabetes (2018) 19(2):305–13. doi: 10.1111/pedi.12563 28745034

[30] Jonsdottir B Larsson C Carlsson A Forsander G Ivarsson SA Lernmark Å. Thyroid and Islet Autoantibodies Predict Autoimmunethyroid Diseaseat Type 1 Diabetes Diagnosis. J Clin Endocrinol Metab (2017) 1;102(4):1277–85. doi: 10.1210/jc.2016-2335.PMID:28388722 PMC5460724 28388722

